# Implementing Oral Antibiotics for Bone and Joint Infections: Lessons Learned and Opportunities for Improvement

**DOI:** 10.1093/ofid/ofae683

**Published:** 2024-11-16

**Authors:** Marten R Hawkins, Elizabeth Thottacherry, Prerak Juthani, Jenny Aronson, Amy Chang, Derek F Amanatullah, Jessie Markovits, Sa Shen, Marisa Holubar, Jason R Andrews, Julie Parsonnet, Daisuke Furukawa

**Affiliations:** Division of Infectious Diseases and Geographic Medicine, Stanford University School of Medicine, Stanford, California, USA; Division of Infectious Diseases and Geographic Medicine, Stanford University School of Medicine, Stanford, California, USA; Department of Internal Medicine, Stanford University School of Medicine, Stanford, California, USA; Division of Infectious Diseases and Geographic Medicine, Stanford University School of Medicine, Stanford, California, USA; Division of Infectious Diseases and Geographic Medicine, Stanford University School of Medicine, Stanford, California, USA; Department of Orthopaedic Surgery, Stanford University School of Medicine, Stanford, California, USA; Department of Internal Medicine, Stanford University School of Medicine, Stanford, California, USA; Quantitative Sciences Unit, Stanford University, Stanford, California, USA; Division of Infectious Diseases and Geographic Medicine, Stanford University School of Medicine, Stanford, California, USA; Division of Infectious Diseases and Geographic Medicine, Stanford University School of Medicine, Stanford, California, USA; Division of Infectious Diseases and Geographic Medicine, Stanford University School of Medicine, Stanford, California, USA; Department of Epidemiology and Population Health, Stanford University, Stanford, California, USA; Division of Infectious Diseases and Geographic Medicine, Stanford University School of Medicine, Stanford, California, USA

**Keywords:** bone and joint infections, oral antibiotics, osteomyelitis, periprosthetic joint infections, guideline implementation

## Abstract

**Background:**

Although intravenous antibiotics have historically been the standard of care for bone and joint infections, clinical trial data have highlighted the safety and efficacy of oral antibiotics. Despite this, intravenous antibiotics are still commonly used, and evaluations of institutional guidelines advancing oral antibiotic use are limited.

**Methods:**

In April 2023, we implemented a new institutional guideline to preferentially treat patients with bone and joint infections with oral antibiotics. The postguideline cohort was compared with a historical preguideline cohort via retrospective chart review. The primary outcome was the proportion of patients discharged exclusively on oral antibiotics. Secondary outcomes included 90-day treatment failure, length of stay, and adverse effects.

**Results:**

One hundred eighty-six patients (53 preguideline and 133 postguideline) were included in the analysis. Patients in the postguideline cohort were more likely to be discharged exclusively on oral antibiotics (25% vs 70%; *P* < .01), with no difference in 90-day treatment failure (8% vs 9%; *P* = .75). Patients in the postguideline cohort had a shorter length of stay than preguideline (median, 8 vs 7 days; *P* = .04) and trended toward fewer peripherally inserted central catheter–related adverse events (6% vs 1%; *P* = .07).

**Conclusions:**

An institutional guideline was effective in increasing the proportion of patients with bone and joint infections discharged on oral antibiotics. We observed similar clinical outcomes after implementing the guidelines while reducing length of hospital stay.

Bone and joint infections are a complicated class of infections. They often require multidisciplinary care by teams of specialists as well as procedural intervention and prolonged durations of antibiotics that are often given intravenously (IV) [1–3]. Despite the historical use of IV antibiotics for these infections, little evidence supports this practice, and guidelines do not even specify the need for prolonged IV therapy [1–5]. Further, the use of IV antibiotics is associated with significant challenges and costs, including the need for central catheters and clinical, biochemical, and drug level monitoring. Central catheters are also associated with risk of infection and thrombus and mechanical malfunction and require skilled nursing support, either in a skilled nursing facility or with home health services at home [6].

Recent literature, particularly the Oral Versus Intravenous Antibiotics for Bone and Joint Infection (OVIVA) trial [7], has demonstrated that the use of oral (PO) antibiotics is safe and effective for the treatment of bone and joint infections. This study also found a significant decrease in length of stay and intravenous catheter complications in those who received PO compared with IV antibiotics. Additionally, a systematic review of randomized controlled trials comparing PO with IV therapy for osteomyelitis found no studies in which IV antibiotics were superior to PO therapy [8].

Despite the literature base supporting the use of PO therapy for bone and joint infections, there are few real-world examples of institutions implementing the findings of the OVIVA trial. Further, many providers are still reluctant to use PO antibiotics for definitive therapy for osteoarticular infections [9]. At our institution, we created a guideline aiming to change practice to preferentially treat patients with bone and joint infections with PO rather than IV antibiotic therapy. The objective of our study was to evaluate the impact of these guidelines on oral antibiotic utilization and clinical outcomes for patients with bone and joint infections.

## METHODS

### Setting

This study was performed at a 706-bed tertiary university teaching hospital in California. Our hospital is a referral center for complex bone and joint conditions with an active orthopedic surgery department. Our hospital has dedicated infectious diseases consult services based on the primary admitting team's specialty (medical, surgical, or intensive care). Most patients with bone and joint infections are seen by the surgical infectious diseases consult service, but some patients, such as those with diabetic foot infections or vertebral osteomyelitis, are admitted to a medicine team and seen by the medical infectious disease service.

### Implementation

In 2023, 4 infectious diseases physicians who specialize in musculoskeletal infections at our institution (E.T., J.A., A.C., D.F.) created a treatment guidance document encouraging the use of PO antibiotics for bone and joint infections (Supplementary Figure 1). This guidance document was created in cooperation with orthopedic surgeons, infectious diseases pharmacists, and the antimicrobial stewardship program. The guideline specifically elucidated criteria for an appropriate switch to PO antibiotics and also recommended specific PO agents for common organisms stratified by the level of published data supporting their use. We presented the guideline to our orthopedic surgery, vascular surgery, and internal medicine colleagues to gain consensus among the services most commonly caring for patients with bone and joint infections.

This treatment guideline was formally introduced on April 1, 2023. Starting this date, the providers on the inpatient infectious diseases services were encouraged to incorporate the guidance document into their practice, and subsequently e-mail reminders about this initiative were sent to fellows and attendings rotating on the inpatient services. Decisions on antibiotics were ultimately left to the discretion of the inpatient providers, and the research team was not directly involved with patient care unless they were, themselves, attending on the inpatient service.

### Patient Cohorts

Both the pre- and postguideline cohorts included patients age 18 years or older who were admitted for bone and joint infections. A database of patients with bone and joint infections was created after introduction of the guideline, and patients were prospectively added to this database as they were seen by the inpatient infectious diseases services. The postguideline cohort included eligible patients in this database who had been discharged between 4/1/2023 and 9/30/2023. The preguideline cohort was queried from our institutions’ clinical data warehouse through the STAnford medicine Research data Repository (STARR) platform using International Classification of Diseases, 10th Edition (ICD-10), codes reflecting bone and joint infections (M86, T84.5, T84.6, T84.7, M46.2, M46.3, M46.4, M46.5, G06.1, M00, M01). Patients were included in the preguideline cohort if they were discharged in the 1-year period before introduction of the guideline. Exclusion criteria for both cohorts included individuals who had no inpatient infectious diseases consultation note, were seen by the immunocompromised host infectious diseases services (typically patients with hematologic malignancy, solid organ transplant, or significant iatrogenic immunosuppression), were treated with antibiotics for <2 weeks, had concomitant infections requiring IV antimicrobial therapy (eg, *Staphylococcus aureus* bacteremia), did not have any outpatient infectious diseases follow-up, had mycobacterial or atypical bacterial infections (eg, *Coxiella* spp.), had decubitus ulcer–associated infections, or had infections of bones and joints outside the spine and extremities (eg, skull-base osteomyelitis). Patients in the postguideline cohort were also excluded if they did not have an encounter associated with the ICD-10 codes used to identify the preguideline cohort.

The primary outcome was the proportion of patients who were discharged on PO antibiotics and no IV antibiotics. Secondary outcomes included the proportion of patients meeting criteria for PO therapy, 90-day clinical failure as defined by the OVIVA trial [7], repeat surgery, re-admission or re-presentation to the emergency department (ED), duration of IV antibiotics, peripherally inserted central catheter (PICC) complications (deep vein thrombosis, line infection, dermatitis, malfunction requiring exchange), length of stay, and discharge location. Patients were considered to meet criteria for PO antibiotics if all of the following criteria were met: (1) culture data showed in vitro susceptibility to PO antibiotics; (2) patients could reliably absorb PO antibiotics; (3) patients did not have a known allergic reaction to appropriate PO antibiotic options; and (4) patients had no concomitant infections for which IV antibiotics were preferred at the discretion of the attending infectious disease consultant. For the purposes of this study, culture-negative infections were considered to not meet criteria for PO antibiotics.

Trained reviewers (M.R.H., P.J., E.T., D.F.) abstracted data from medical records, including outcomes, demographics, comorbidities, infection subtype, antibiotic course, and microbiologic results. In-depth chart review was also performed for all patients with 90-day failure who were discharged on PO therapy in an effort to determine etiologies of failure. All data were recorded in REDCap. Within the postguideline cohort, we also examined primary and secondary outcomes among patients discharged on IV antibiotics in comparison with patients discharged on PO antibiotics. Both cohorts were followed for 90 days from time of discharge.

### Statistical Analysis

Categorical variables were summarized by frequency and proportion, and continuous variables were summarized by median and interquartile range. For any 2-group comparison, a 2-proportions z-test was used for categorical variables, and the Wilcoxon rank-sum test was used for continuous variables. The Fisher exact test was used for categorical variables when the assumptions of the z-test were not met. Analyses were not corrected for multiple testing to ensure that all possibly important differences were observed.

Bivariate analysis was performed to examine potential predictors of patients discharging on oral antibiotics. For continuous variables, group comparison was conducted using the Wilcoxon rank-sum test, and for categorical variables the chi-square test and Fisher exact test were conducted. The standardized mean difference (SMD) was calculated to evaluate the absolute difference and variable balance between the 2 groups. An SMD of 0.2 to 0.5 indicated a small imbalance, 0.5 to 0.8 medium, and >0.8 large [10]. Potential predictors were selected to be included in the subsequent analysis according to the results of the bivariate analysis (*P* < .10) and theory. A modified multivariable Poisson regression model was used to test the effect of the intervention (guideline implementation), adjusting for identified factors that might have influenced antibiotic choice. Crude relative risks, adjusted relative risks, and their corresponding 95% confidence intervals were estimated by Poisson regression using robust error variance [11].

Analysis was performed using SAS, version 9.4 (SAS Institute Inc.), for all analyses. All statistical tests were evaluated at a 2-sided alpha level of .05. The institutional review board at our institution approved this study (protocol #67967) with waived consent.

## RESULTS

One hundred eighty-six patients, 53 in the preguideline cohort and 133 in the postguideline cohort, were included in the study. The pre- and postguideline cohorts had similar baseline demographic and clinical characteristics (Table 1); however, there were more patients with autoimmune disease (15% vs 2%; *P* < .01) and coagulase-negative *Staphylococcus* spp. infections (38% vs 19%; *P* = .01) in the preguideline cohort. Both cohorts had a large proportion of patients with polymicrobial infections, with a trend toward a higher proportion in the preguideline cohort (85% vs 74%; *P* = .10). Both populations had a high frequency of previous joint infection and infections related to implants. Within the postguideline cohort, patients discharged on PO and IV antibiotics were also similar at baseline, although a higher proportion of patients who received IV antibiotics had vertebral osteomyelitis (9% vs 32%; *P* < .01) and a lower proportion had diabetic foot infections (26% vs 10%; *P* = .06) compared with patients on PO. Given low SMD values across variables between cohorts, the cohorts were deemed similar and balanced.

**Table 1.** Baseline Characteristics

					Postguideline			
	Preguideline	Postguideline	*P* Value	SMD	IV	PO		
	No. (%)/Median (IQR)	No. (%)/Median (IQR)			No. (%)/Median (IQR)	No. (%)/Median (IQR)	*P* Value	SMD
Total	53	133			40	93		
Age, y	65.9 (57.3–74.4)	68.24 (52.8–77.5)	.65	0.06	67.9 (57.4–79.0)	68.2 (48.7–76.6)	.41	0.26
Male	33 (62)	77 (58)	.58	0.09	25 (62)	52 (56)	.13	0.48
Body mass index, kg/m^2^	26 (24.1–31.8)	27.8 (24.6–32)	.30	0.14	26.9 (24.4–32.2)	28.1 (24.7–32)	.72	0.05
Medical history								
Diabetes mellitus	19 (36)	57 (43)	.38	0.14	17 (43)	40 (43)	.96	0.01
Coronary artery disease	12 (23)	36 (27)	.53	0.1	12 (30)	24 (25)	.62	0.09
Chronic kidney disease	4 (8)	24 (18)	.11	0.32	10 (25)	14 (15)	.17	0.25
Cirrhosis	1 (2)	6 (5)	.68	0.15	3 (8)	3 (3)	.36	0.19
Peripheral artery disease	5 (9)	23 (17)	.26	0.23	7 (18)	16 (17)	.97	0.01
Autoimmune disease	8 (15)	2 (2)	<.01	0.51	1 (3)	1 (1)	.51	0.11
Malignancy	3 (6)	13 (10)	.56	0.15	4 (10)	9 (10)	1	0.01
Immunosuppressed	4 (8)	9 (7)	1.00	0.03	3 (8)	6 (6)	1	0.04
Smoking	5 (9)	10 (8)	.77	0.07	2 (5)	8 (9)	.72	0.14
History of infection, same joint	19 (36)	37 (28)	.28	0.17	13 (33)	24 (26)	.43	0.15
History of infection, different joint	7 (13)	16 (12)	.83	0.04	5 (13)	11 (12)	1	0.02
Type of infection								
Prosthetic joint infection	22 (42)	47 (35)	.43	0.13	18 (44)	29 (31)	.16	0.26
Diabetic foot	13 (25)	28 (21)	.60	0.08	4 (10)	24 (26)	.06	0.43
Vertebral osteomyelitis/discitis	9 (17)	21 (16)	.84	0.03	13 (32)	8 (9)	.001	0.60
Native joint/long bone	5 (9)	27 (20)	.09	0.31	4 (10)	23 (25)	.06	0.40
Hardware infection	4 (8)	11 (8)	1.00	0.03	1 (2)	10 (11)	.17	0.34
Microbiology								
Negative	17 (32)	35 (26)	.43	0.13	14 (35)	21 (23)	.14	0.28
*Staphylococcus aureus*	13 (25)	35 (26)	.60	0.09	10 (25)	25 (27)	.82	0.04
Coagulase-negative *Staphylococcus*	20 (38)	25 (19)	.01	0.43	5 (13)	20 (22)	.33	0.24
*Streptococcus* spp.	8 (15)	16 (12)	.57	0.09	4 (10)	12 (13)	.78	0.09
*Enterococcus* spp.	5 (9)	8 (6)	.52	0.13	3 (8)	5 (5)	.7	0.09
*Pseudomonas aeruginosa*	7 (13)	8 (6)	.10	0.25	4 (10)	4 (4)	.24	0.22
Other gram-negative rods	4 (8)	10 (8)	1.00	0	1 (3)	9 (10)	.28	0.30
*Cutibacterium acnes*	0 (0)	3 (2)	.56	0.22	0 (0)	3 (3)	.55	0.26
*Candida* spp.	3 (6)	3 (2)	.35	0.18	0 (0)	3 (3)	.55	0.26
Other^a^	15 (28)	23 (17)	.05	0.31	10 (25)	13 (14)	.12	0.28
Polymicrobial	45 (85)	98 (74)	.10	0.28	29 (73)	69 (74)	.84	0.04
Bacteremia	6 (11)	5 (4)	.08	0.29	2 (5)	3 (3)	.63	0.64
Type of surgery								
No surgery	7 (13)	19 (14)	.85	0.03	5 (12)	14 (15)	.79	0.08
Debridement	13 (25)	32 (24)	.95	0.01	7 (17)	25 (27)	.22	0.24
Amputation	9 (17)	16 (12)	.37	0.14	2 (5)	14 (15)	.15	0.34
Implant present and retained	12 (23)	21 (16)	.27	0.17	8 (20)	13 (14)	.42	0.15
Implant present and removed	13 (25)	29 (22)	.69	0.06	7 (17)	22 (24)	.4	0.16
Implant present and exchanged	1 (2)	11 (8)	.18	0.29	6 (15)	5 (5)	.09	0.31
Implant not present and placed	1 (2)	9 (7)	.29	0.24	4 (10)	5 (5)	.45	0.17
Spacer exchange	3 (6)	7 (5)	1.00	0.02	4 (10)	3 (3)	.2	0.27
Antibiotic cement/bead used	13 (25)	38 (29)	.58	0.09	12 (29)	26 (28)	.88	0.03
Systemic antibiotics								
Discharged exclusively on orals	13 (25)	93 (70)	<.01	1.02	NA	NA	NA	NA
Met criteria for orals	37 (70)	80 (60)	.22	0.2	17 (43)	63 (68)	<.01	
Duration of IV antibiotics, d	45 (22–53)	7 (4–28)	<.01	0.96	44 (35–56.5)	5 (3–5)	<.01	2.99
Duration of total antibiotics, d	65.5 (45–90)	49 (42–90)	.04	0.35	61.5 (44–90)	45 (35–75)	.02	0.48

Abbreviations: IQR, interquartile range; IV, intravenous; PO, oral; SMD, standardized mean difference.

^a^ *Corynebacterium* spp. (n = 12), anaerobes (n = 11), mold (n = 3), *Bacillus* spp. (n = 2), *Gamella morbillorum* (n = 2), *Micrococcus* (n = 2), unidentified gram-positive (n = 2), *Neisseria gonorrhea* (n = 1), *Pasteurella multicoda* (n = 1), *Actinomyces* (n = 1), *Haemophilus influenzae* (n = 1), *Rothia* (n = 1).

In the preguideline cohort, 25% (n = 13) of patients were discharged on PO antibiotics compared with 70% (n = 93; *P* < .01) of patients in the postguideline cohort despite similar proportions of patients meeting criteria for PO antibiotics. Patients in the postguideline cohort had a significantly shorter duration of IV antibiotics (median, 45 vs 7 days; *P* < .01) and a shorter total duration of antibiotic therapy (median, 66 days vs 49 days; *P* = .04) compared with patients in the preguideline cohort (Table 1). Data on specific antibiotics prescribed on discharge are presented in Supplementary Table 1. IV antimicrobials, including penicillin-class antibiotics, cephalosporins, and daptomycin, were used more often in the preguideline cohort, whereas PO antibiotics, including oral penicillin-class antibiotics, fluoroquinolones, doxycycline, and trimethoprim-sulfamethoxazole, were more common in the postguideline cohort.

Of the patients who met criteria for PO antibiotics, there was a significant increase in the proportion of patients who were discharged on PO antibiotics compared with the preguideline cohort (27% vs 79%; *P* < .01) (Table 2). Although numbers were small, similar trends were seen in all subtypes of bone and joint infections. Reasons for discharge on IV antibiotics despite meeting criteria for PO included surgeon preference (n = 5), concern for oral side effects (n = 3), concern for lack of source control (n = 3), complicated history (n = 2), bacteremia (n = 2), and unclear from chart review (n = 3). Among patients who did not meet criteria for PO antibiotics, 3 of 16 (19%) in the preguideline cohort and 30 of 53 (57%) in the postguideline cohort were discharged on PO therapy (*P* = .01).

**Table 2.** Proportion of Patients who Met Criteria for PO Antibiotics who Were Discharged on PO

	Preguideline, n/N (%)	Postguideline, n/N (%)	*P* Value
Total	10/37 (27)	63/80 (79)	<.01
Diabetic foot	7/9 (78)	15/16 (94)	.50
Native joint/long bone	2/3 (67)	15/17 (88)	.40
Hardware infection	1/4 (25)	7/8 (88)	.07
Prosthetic joint infection	0/16 (0)	20/28 (71)	<.01
Vertebral osteomyelitis/discitis	0/5 (0)	6/11 (55)	.09

Abbreviation: PO, oral.

In the multivariable analysis, being in the postguideline cohort was an independent predictor of discharging on oral antibiotics (adjusted relative risk [aRR], 2.62; 95% CI, 1.7–4.01) (Table 3). Additionally, patients meeting criteria for PO antibiotics (aRR, 1.36; 95% CI, 1.06–1.76) were more likely to be discharged on PO antibiotics. Conversely, patients with prosthetic joint infection (aRR, 0.65; 95% CI, 0.46–0.92) and vertebral osteomyelitis/discitis (aRR, 0.43; 95% CI, 0.23–0.82) were less likely to receive PO antibiotics.

**Table 3.** Predictors of Patients Discharged on PO Antibiotics

	Discharged on PO Antibiotics	Discharged on IV Antibiotics	*P* Value		*P* Value		*P* Value
	No. (%)/Median (IQR)	No. (%)/Median (IQR)		Crude RR (95% CI)		Adjusted RR (95% CI)	
Total	106	80		…	…	…	…
Age, y	67.4 (48.7–76.4)	66.7 (57.4–44.8)	.37	…	…	…	…
Male	59 (56)	51 (64)	.27	…	…	…	…
Body mass index, kg/m^2^	27.8 (23.9–31.5)	26.8 (24.5–32.4)	.99	…	…	…	…
Medical history	…	…		…	…	…	…
Diabetes mellitus	48 (45)	28 (35)	.16	…	…	…	…
Coronary artery disease	26 (25)	22 (28)	.65	…	…	…	…
Chronic kidney disease	15 (14)	13 (16)	.69	…	…	…	…
Cirrhosis	3 (3)	4 (5)	.47	…	…	…	…
Peripheral artery disease	19 (18)	9 (11)	.21	…	…	…	…
Autoimmune disease	3 (3)	7 (9)	.10	…	…	…	…
Malignancy	9 (8)	7 (9)	.95	…	…	…	…
Immunosuppressed	7 (7)	6 (8)	.81	…	…	…	…
Smoking	11 (10)	4 (5)	.28	…	…	…	…
History of infection same joint	26 (25)	30 (38)	.06	0.73 (0.54–0.99)	.05	0.86 (0.67–1.1)	.23
History of infection different joint	15 (14)	8 (10)	.39	…	…	…	…
Type of infection	…	…		…	…	…	…
Prosthetic joint infection	29 (27)	40 (50)	<.01	0.64 (0.47–0.86)	<.03	0.65 (0.46–0.92)	0.02
Diabetic foot	32 (30)	9 (11)	<.01	1.51 (1.17–1.94)	<.01	1.18 (0.82–1.7)	.37
Vertebral osteomyelitis/discitis	8 (8)	22 (28)	<.01	0.43 (0.23–0.79)	.01	0.43 (0.23–0.82)	.01
Native joint/long bone	27 (26)	5 (6)	<.01	1.65 (1.33–2.05)	<.01	1.2 (0.88–1.63)	.24
Hardware infection	11 (10)	4 (5)	.28	…	…	…	…
Microbiology	…	…		…	…	…	…
Negative	25 (24)	27 (34)	.13	…	…	…	…
*Staphylococcus aureus*	29 (27)	18 (23)	.45	…	…	…	…
Coagulase-negative *Staphylococcus*	26 (25)	19 (24)	.90	…	…	…	…
*Streptococcus* spp.	15 (14)	9 (11)	.56	…	…	…	…
*Enterococcus* spp.	6 (6)	7 (9)	.41	…	…	…	…
*Pseudomonas aeruginosa*	6 (6)	9 (11)	.17	…	…	…	…
Other gram-negative rods	10 (9)	4 (5)	.4	…	…	…	…
*Cutibacterium acnes*	3 (3)	0 (0)	.26	…	…	…	…
*Candida* spp.	5 (5)	1 (1)	.24	…	…	…	…
Other^a^	17 (16)	22 (28)	.06	0.74 (0.51–1.08)	.12	0.72 (0.5–1.02)	.06
Polymicrobial	80 (75)	63 (79)	.6	…	…	…	…
Bacteremia	3 (3)	8 (10)	.06	0.44 (0.16–1.26)	.13	0.9 (0.39–2.06)	.8
Type of surgery	…	…		…	…	…	…
No surgery	16 (15)	10 (13)	.64	…	…	…	…
Debridement	30 (28)	15 (19)	.17	…	…	…	…
Amputation	19 (18)	6 (8)	.06	1.41 (1.07–1.85)	.01	1.07 (0.74–1.53)	.72
Implant present and retained	13 (12)	20 (25)	.04	0.63 (0.39–1.01)	.06	0.98 (0.65–1.48)	.94
Implant present and removed	23 (22)	19 (24)	.76	…	…	…	…
Implant present and exchanged	5 (5)	7 (9)	.37	…	…	…	…
Implant not present and placed	2 (2)	4 (5)	.4	…	…	…	…
Spacer exchange	4 (4)	6 (8)	.33	…	…	…	…
Antibiotic cement/bead used	28 (26)	23 (29)	.72	…	…	…	…
Met criteria for oral antibiotics	73 (69)	44 (55)	.05	1.31 (0.99–1.72)	.06	1.36 (1.06–1.76)	.02
Postguideline	93 (88)	40 (50)	<.01	2.84 (1.75–4.62)	<.01	2.62 (1.7–4.01)	<.01

Abbreviations: IQR, interquartile range; IV, intravenous; PO, oral; RR, relative risk.

Secondary complications, including need for infection-related repeat surgery, repeat ED presentation, readmission, antibiotic adverse events, and death, were similar between both cohorts. Median length of hospital stay was shorter in the postguideline cohort (8 days vs 7 days; *P* = .04), with trends toward increased discharge to home and fewer PICC-related adverse events in the postguideline cohort (Table 4). In the postguideline cohort, the median length of stay was shorter in the PO group compared with IV (9 days vs 7 days; *P* = .01), but there were no differences in rates of other secondary outcomes between these 2 postguideline groups.

**Table 4.** Clinical Outcomes and Secondary Measures

				Postguideline		
	Preguideline, No. (%)/Median (IQR)	Postguideline, No. (%)/Median (IQR)	*P* Value	IV	PO	*P* Value
				No. (%)/Median (IQR)	No. (%)/Median (IQR)	
Total	53	133	…	40	93	…
Clinical outcomes measures						
Treatment failure	4 (8)	12 (9)	.75	1 (2)	11 (12)	.07
Repeat surgery	6 (11)	26 (20)	.18	6 (15)	20 (22)	.32
ED presentation	2 (4)	10 (8)	.51	3 (7)	7 (8)	1.00
Readmission	7 (13)	33 (25)	.08	9 (22)	24 (26)	.64
Death	1 (2)	4 (3)	1.00	2 (5)	2 (2)	.58
Length of stay, d	8 (5–13)	7 (4–11)	.04	9 (6–12)	7 (4–10)	.01
PICC-related adverse events	3 (6)	1 (1)	.07	1 (2)	0 (0)	.30
Discharge to home	29 (55)	89 (66)	.14	24 (59)	65 (70)	.20
Antibiotic adverse events^a^	6 (11)	25 (19)	.22	10 (24)	15 (16)	.26
Mild	4 (67)	15 (60)	1.00	4 (40)	11 (73)	.12
Serious	2 (33)	10 (40)	1.00	6 (60)	4 (27)	.12

Abbreviations: ED, emergency department; IQR, interquartile range; IV, intravenous; PICC, peripherally inserted central catheter; PO, oral.

^a^Mild = rash/pruritis, gastrointestinal intolerance. Serious was defined as *Clostridioides difficile* infection, acute kidney injury, hepatitis, cytopenia, neurotoxicity, other severe allergies (eg, drug rash eosinophilia systemic symptoms [DRESS]).

Ninety-day failure rates were low and did not differ significantly in the pre- and postguideline cohorts (8% preguideline vs 9% postguideline; *P* = .76) (Table 4). Within the postguideline cohort only, however, there was a tendency toward a higher failure rate in patients who were discharged on PO antibiotics compared with those discharged on IV antibiotics (12% vs 3%; *P* = .08).

Of the 11 total failures in the PO arm of the postguideline cohort, 7 occurred in patients with diabetic foot infection, 3 in patients with prosthetic joint infection, and 1 in a patient with native joint septic arthritis. Clinical characteristics of each of these cases are summarized in Table 5. All patients who failed treatment had high-risk comorbidities, including 7 who had both peripheral arterial disease and diabetes mellitus. Two patients were found to have poor adherence to therapy, and 2 were prescribed antibiotics that did not have definite in vitro activity against the organism recovered. These 2 were both patients with prosthetic joint infections who had coagulase-negative *Staphylococcus* recovered on culture, but susceptibilities were not performed on the initial isolates. They then experienced treatment failure and underwent repeat operative intervention with operative cultures again growing coagulase-negative *Staphylococcus* isolates, with susceptibility testing performed that showed resistance to the initial PO treatment regimen. The 1 failure in the IV group was also of a patient with diabetic foot infection with comorbidities including peripheral artery disease and end-stage renal disease.

**Table 5.** Summary of Patients who Experienced Treatment Failure on PO Antibiotics

Infection Type	Body Part	Type of Surgery	Organisms	Antibiotics	Risk Factors
Diabetic foot infection	Foot	I&D	*Pseudomonas aeruginosa*	Ciprofloxacin, cefadroxil	Host factors: DM, PAD, immunosuppression
Diabetic foot infection	Foot	I&D	*Corynebacterium striatum*	Amoxicillin-clavulanate −> linezolid	Host factors: DM, PAD
Diabetic foot infection	Foot	Ray amputation	*Proteus mirabilis*	Amoxicillin-clavulanate	Host factors: DM, PAD
Diabetic foot infection	Foot	Transmetatarsal amputation	*Streptoccus agalactiae, Enterococcus faecalis, Staphyloccocus lugdunensis*	Amoxicillin-clavulanate	Host factors: DM, PAD, ESRD
Diabetic foot infection	Foot	None	None	Amoxicillin-clavulanate + doxycycline	Host factors: DM, PAD, ESRD, obesity
Diabetic foot infection	Foot	Partial ray amputation	MSSA*, Bacteriodies fragilis*, anaerobic GPC	Amoxicillin-clavulanate	Host factors: DM, PAD Medication nonadherence
Diabetic foot infection	Foot	None	None	Amoxicillin-clavulanate + doxycycline	Host factors: DM, PAD, ESRD
Prosthetic joint infection	Hip	Implant present and retained	*Enterococcus faecalis*	Linezolid	Host factors: obesity, history of infection of same joint, history of radiation, tumor prosthesis, implant retention
Prosthetic joint infection	Shoulder	Implant removal and antibiotic cement placement	Coagulase-negative *Staphylococcus*	Doxycycline	Host factors: obesity Possible bug–drug mismatch
Prosthetic joint infection	Knee	Implant present and retained	*Corynebacterium striatum*, coagulase negative *Staphylococcus,*	Doxycycline + cefadroxil	Host factors: obesity, poor wound healing, implant retention Possible bug–drug mismatch
Septic arthritis	Knee	I&D	MSSA	Trimethoprim-sulfamethaxozole	Host factors: smoking Medication nonadherence

Abbreviations: DM, diabetes mellitus; ESRD, end-stage renal disease; GPC, gram-positive cocci; I&D, incision and drainage; MSSA, methicillin-susceptible *Staphylococcus aureus*; PAD, peripheral artery disease.

## DISCUSSION

Our study supports the efficacy of an institutional guideline to preferentially discharge patients on PO antibiotics for bone and joint infections. Overall, uptake of oral antibiotics was excellent, with IV antibiotics reserved for reasonable clinical situations. Importantly, we demonstrated comparable outcomes between the preguideline and postguideline cohorts, adding to the body of literature supporting that PO therapy can be effectively implemented in this patient population [7, 8, 12–14]. Additionally, overall duration of antibiotics did not increase in the postguideline cohort, suggesting that clinicians did not increase the use of suppression or extend courses due to concern for failure.

The increase in patients discharged on PO therapy led to other important benefits. Patients in the postguideline cohort had a decreased length of stay compared with those in the preguideline cohort. The postguideline cohort also showed a trend toward more discharges to home and reduced PICC line complications, thereby likely improving patient safety and satisfaction [15, 16]. Past studies have also shown significant cost savings with implementation of PO antibiotics [14, 17], and, though not formally calculated, with reduction in length of stay, days of IV antibiotics, and resource utilization such as nursing homes and home health services, we expect similar cost savings in our cohort of patients.

We also note areas for future improvement. Patients with vertebral osteomyelitis and prosthetic joint infections were less likely to be discharged on PO antibiotics. The reluctance to treat these conditions with PO antibiotics is consistent with what is observed nationally. In a nationally conducted survey, only 22% and 28% of respondents reported using oral antibiotics as definitive therapy for vertebral osteomyelitis and orthopedic hardware infections, respectively [9]. It is important to note that this lack of PO antibiotic utilization is not due to lack of evidence given that recent guidelines, including those for vertebral osteomyelitis and hardware infections, are now specifically recommending PO antibiotics [5, 18, 19]. Additional outreach efforts are needed to better educate providers in order to further facilitate guideline-concordant practices.

Though not statistically significant, in the postimplementation cohort there were more treatment failures in the PO group compared with the IV group, which were driven primarily by patients with diabetic foot infections. Diabetic foot infections are complex and difficult to treat, requiring multidisciplinary care [20]. While antibiotics are frequently used, they may not be the most important factor in determining risk of relapse [21]. All diabetic foot patients who failed in our study also had peripheral vascular disease, which may have not only affected drug delivery but also likely predisposed them to wound complications and overall risk of infection relapse [22]. Additionally, the majority of failures in diabetic foot infections in our cohort were patients treated with amoxicillin-clavulanate. Though these failures may have been due to factors outside of antibiotic efficacy as discussed above, experts have questioned oral beta-lactam's ability to attain pharmacokinetic-pharmacodynamic targets against gram-negative infections [23]. High doses of amoxicillin (1 g 3 or 4 times a day) are thus suggested for oral transition therapy [5, 23, 24], and the traditional twice-daily dosing for amoxicillin-clavulanate, which was used for our patients, may be inadequate for osteoarticular infections. Further studies are needed to better evaluate the efficacy of oral beta-lactams for osteoarticular infections.

There were other notable observations from reviewing patients who failed on PO therapy. Of the 3 patients with prosthetic joint infections who failed, 2 had potential selection of an inactive antibiotic (ie, bug–drug mismatch), and the third had significant comorbidities including radiation history and a large tumor prosthesis that was retained, which may have increased risk of failure. The 1 failure among septic arthritis cases was in a patient with an unstable social situation and documented nonadherence to prescribed antibiotics, which illustrates a situation where ongoing IV antibiotics in a monitored setting or, alternatively, long-acting IV antibiotics may have been beneficial. These results highlight the importance of carefully considering host and microbiologic factors when choosing an antimicrobial regimen for these patients. Future studies are needed to determine if patients with specific host factors, pathogens, infection type, or combinations of factors may benefit from IV therapy over PO.

Our study has several limitations. First, this was a single-center retrospective study with a relatively low number of patients evaluated. Second, the 2 cohorts were identified from 2 separate databases, and thus the cohorts may not have been truly comparable. However, to address this potential issue, we provided extensive baseline characteristics of the 2 cohorts, and, reassuringly, there were few statistically significant differences between the 2 cohorts and SMD values were small across group comparisons. Additionally, we ensured that all patients in the postguideline cohort had an encounter with an associated ICD-10 code used to identify the preguideline cohort. Third, like other studies in this area, our study was not designed to compare regimens, and the optimal regimens for different pathogens or types of infection remain unknown. Lastly, our follow-up period of 90 days for evaluation of clinical outcomes was shorter than comparable studies, though this would not have impacted our primary outcome of proportion of patients discharged on PO antibiotics. Future studies with a longer follow-up period are needed to better evaluate the long-term efficacy of PO antibiotics.

In conclusion, we were able to show that an institutional guideline promoting PO antibiotics for bone and joint infections can be effectively implemented to increase PO antibiotic utilization. Our intervention led to similar clinical outcomes while reducing length of stay and trending toward other improved patient-centered metrics. Our study also shed light on potential factors that may lead to poor outcomes for patients discharged on PO antibiotics. Future studies are needed to better identify circumstances in which patients may be poor candidates for PO therapy, particularly as institutions implement their own criteria for PO therapy for treatment of bone and joint infections.

## Supplementary Material

ofae683_Supplementary_Data

## Supplementary Data

Supplementary materials are available at *Open Forum Infectious Diseases* online. Consisting of data provided by the authors to benefit the reader, the posted materials are not copyedited and are the sole responsibility of the authors, so questions or comments should be addressed to the corresponding author.

## Notes

***Author contributions. *** Study conception: E.T., J.A., A.C., D.F.A., J.M., D.F. Study design: M.R.H., S.S., M.H., J.R.A., J.P., D.F. Data collection: M.R.H., E.T., P.J., D.F. Data analysis: S.S., J.P., D.F. First draft: M.R.H. Critical edits: all authors.
