# Supplementary material for: Implementing Oral Antibiotics for Bone and Joint Infections: Lessons Learned and Opportunities for Improvement
Source: Open Forum Infect Dis. 2024 Nov 16;11(12):ofae683. doi: 10.1093/ofid/ofae683 (PMC11629981; doi:10.1093/ofid/ofae683)
Supplement: ofae683_Supplementary_Data [file ofae683_supplementary_data.docx]

Supplemental Figure 1: Select sections from our bone and joint infection treatment guideline


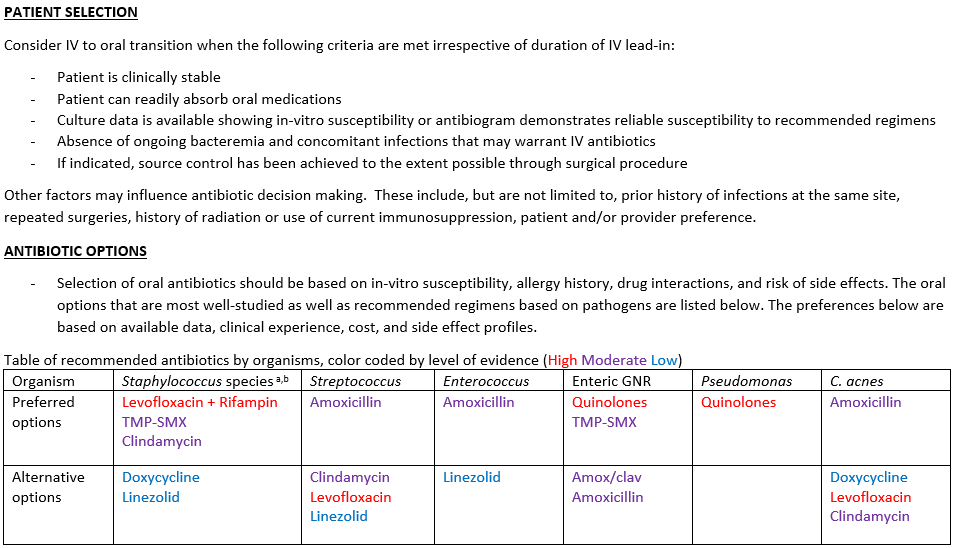


Supplemental Table 1: Comparison of discharge antibiotics between pre-guideline and post-guideline cohorts

|  | Pre-guideline  N (%) / Median (IQR) | Post-guideline  N (%) / Median (IQR) | p-value |
| --- | --- | --- | --- |
| Total | 53 | 133 |  |
| Discharge antibiotics |  |  |  |
| IV cephalosporin | 22 (42) | 25 (19) | <0.01 |
| Vancomycin | 12 (23) | 17 (13) | 0.12 |
| IV penicillin | 6 (11) | 1 (1) | <0.01 |
| Daptomycin | 4 (8) | 1 (1) | 0.02 |
| Carbapenem | 3 (6) | 5 (4) | 0.69 |
| PO penicillin | 6 (11) | 37 (28) | 0.02 |
| Doxycycline | 5 (9) | 32 (24) | 0.03 |
| Quinolones | 3 (6) | 29 (22) | 0.01 |
| Trimethoprim-sulfamethoxazole | 1 (2) | 16 (12) | 0.04 |
| Rifampin | 4 (8) | 14 (11) | 0.78 |
| Other PO^a^ | 2 (4) | 9 (7) | 0.73 |
| Antifungals | 3 (6) | 1 (1) | 0.07 |

^a^ metronidazole (n=6), linezolid (n=3), clindamycin (n=2)
